# The Underutilization of Urea in the Treatment of SIADH – An Efficacious but Overlooked Solution

**DOI:** 10.7759/cureus.74265

**Published:** 2024-11-22

**Authors:** FNU Sorath

**Affiliations:** 1 Anaesthesia, Dow Health Sciences Karachi, Karachi, PAK

**Keywords:** hyponatremia, siadh, syndrome of inappropriate secretion of antidiuretic hormone (siadh), treatment of hyponatremia, urea

## Abstract

The syndrome of inappropriate antidiuretic hormone secretion (SIADH) is a frequent cause of hyponatremia that presents substantial management challenges in clinical settings. Despite a range of treatment options, including fluid restriction, demeclocycline, and vasopressin antagonists, urea remains underutilized, particularly in North America, despite its well-documented efficacy, safety, and cost-effectiveness. Urea corrects hyponatremia by promoting osmotic diuresis without causing significant fluid shifts, making it an ideal treatment for both acute and chronic SIADH. Comparative studies have demonstrated urea’s effectiveness, particularly in contrast to vasopressin antagonists, which are costly and pose risks such as hepatotoxicity and rapid sodium overcorrection. However, barriers to urea's utilization include limited clinician familiarity, lack of advocacy in guidelines, and patient adherence issues due to its unpalatable taste, although flavored formulations now address this issue. Increased awareness, training, and guideline inclusion could promote urea as a viable, primary treatment for SIADH. This editorial advocates for the expanded adoption of urea in clinical practice to enhance patient outcomes, especially in resource-limited settings where high-cost treatments may not be feasible.

## Editorial

SIADH is a common and challenging clinical condition leading to hyponatremia. When untreated or inadequately managed, it can result in significant morbidity and mortality. While several therapeutic options exist, urea is one of the most effective yet underutilized treatments, particularly in North America, where newer agents such as vasopressin antagonists are more frequently prescribed despite their costs and associated adverse effects. This editorial explores the mechanisms, benefits, and barriers to adopting urea in SIADH treatment and argues for its broader utilization in clinical practice.

Mechanisms and benefits of urea in SIADH

SIADH results in the excessive release of antidiuretic hormone (ADH), leading to water retention, dilutional hyponatremia, and reduced serum osmolality [1]. Urea addresses these issues directly by promoting an osmotic diuresis. Once administered, urea is freely filtered by the kidneys and not reabsorbed, drawing water out and thereby effectively correcting the hyponatremia without leading to significant fluid shifts. This makes urea an ideal treatment for managing both acute and chronic hyponatremia.

Several studies have highlighted urea’s efficacy in treating SIADH. Urea has shown rapid, predictable increases of 5-10 mEq/L of serum sodium levels, reducing the risk of osmotic demyelination syndrome (ODS), a risk with overly rapid correction in hyponatremia [2,3]. Additionally, urea can be administered orally and intravenously, providing flexibility based on the clinical setting and patient need. In Europe, urea has long been a mainstay of SIADH management, underscoring its safety and effectiveness.

Comparison to other treatment options

Commonly used treatments for SIADH, such as fluid restriction, demeclocycline, and vasopressin antagonists, each have their limitations [4]. Though often the first step, fluid restriction is frequently ineffective and poorly tolerated by patients, particularly in cases of severe hyponatremia. Demeclocycline has delayed onset and carries risks of nephrotoxicity, which is concerning in patients with renal impairment [4]. While effective, vasopressin antagonists come with substantial cost burdens and potential adverse effects, including risks of hepatotoxicity and rapid overcorrection of sodium.

Compared to these options, urea is cost-effective and avoids these limitations, providing a safe, effective, and affordable alternative. The economic aspect is crucial in resource-limited settings where high-cost therapies may be inaccessible. Additionally, urea does not require intensive monitoring and dose adjustments, simplifying its use in outpatient settings and long-term management.

Barriers to utilization

Despite its efficacy and safety profile, urea remains underutilized in many countries, including the United States. It may stem from a lack of familiarity among clinicians, limited guidelines advocating its use, and a tendency toward newer, more expensive pharmacologic agents [2,3]. Additionally, urea’s unpalatability in its oral form has been a barrier, as patients often find the taste unpleasant. However, flavored formulations have been developed to improve adherence and tolerability.

Another significant barrier is the lack of large randomized controlled trials comparing urea directly with other treatment modalities despite abundant observational data supporting its efficacy. Greater emphasis on this data within clinical guidelines could increase confidence among healthcare providers in recommending urea for SIADH.

Moving forward: advocating for urea in SIADH treatment

Given its advantages, urea deserves a renewed focus in clinical practice and guidelines for SIADH management. Increased training and awareness about urea's efficacy and safety could help shift clinician perception. Encouragingly, recent cost-effectiveness analyses and reviews have begun to highlight urea as an essential tool, particularly in high-burden settings. Additionally, flavored formulations offer a solution to previous adherence challenges.

Conclusion

Urea is a safe, effective, and cost-efficient treatment for SIADH that has been overlooked in favor of newer, more expensive alternatives. It is time for clinicians and healthcare systems to recognize the value of this established therapy, integrate it into standard treatment protocols, and promote its use where appropriate. The evidence in favor of urea is clear: what remains is the need for a collective effort to bring it back into the mainstream for the benefit of patients suffering from this challenging condition.
